# Supplementary material for: Anti-Thrombogenicity Study of a Covalently-Attached Monolayer on Stent-Grade Stainless Steel
Source: Materials (Basel). 2021 Apr 30;14(9):2342. doi: 10.3390/ma14092342 (PMC8125229; doi:10.3390/ma14092342)
Supplement: Supplementary file 1 [file materials-14-02342-s001.zip › materials-1168988-supplementary.pdf]

materials

Supplementary Material

Anti-Thrombogenicity Study of a Covalently-Attached Monolayer on Stent-Grade Stainless Steel

Tairan Yang, Brian De La Franier and Michael Thompson *

Department of Chemistry, University of Toronto, 80 St. George Street, Toronto, ON M5S 3H6, Canada; tairan.yang@mail.utoronto.ca (T.Y.B.); brian.delafranier@mail.utoronto.ca (B.D.L.F.)
* Correspondence: m.thompson@utoronto.ca; Tel.: +1-416-978-3575

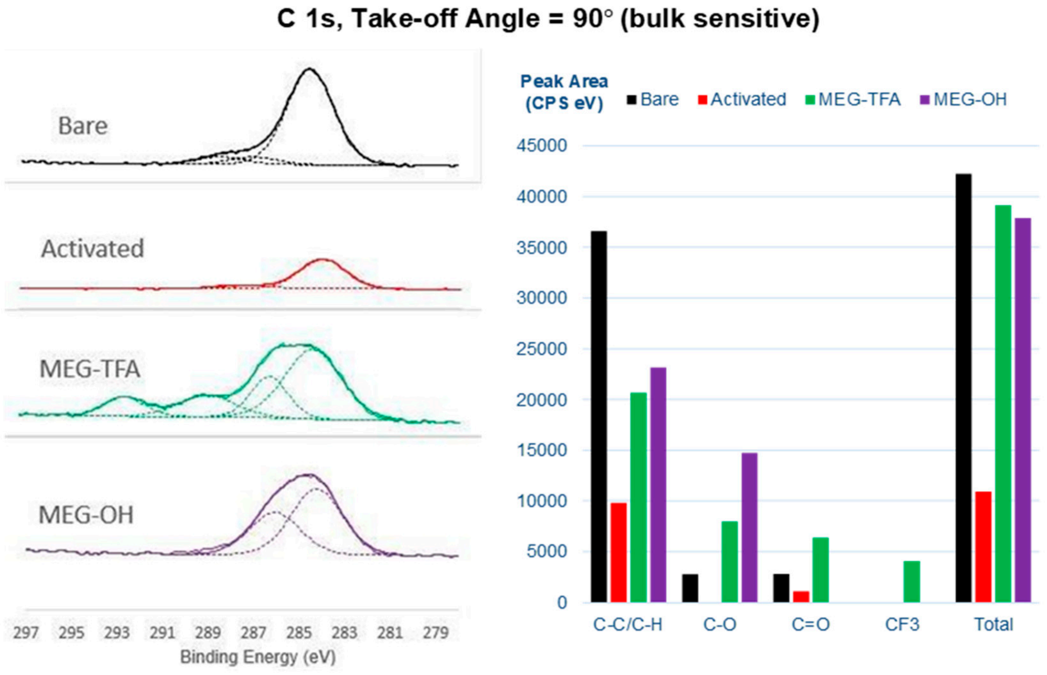

Figure S1. C 1s, Take-off Angle = 90° (bulk sensitive).

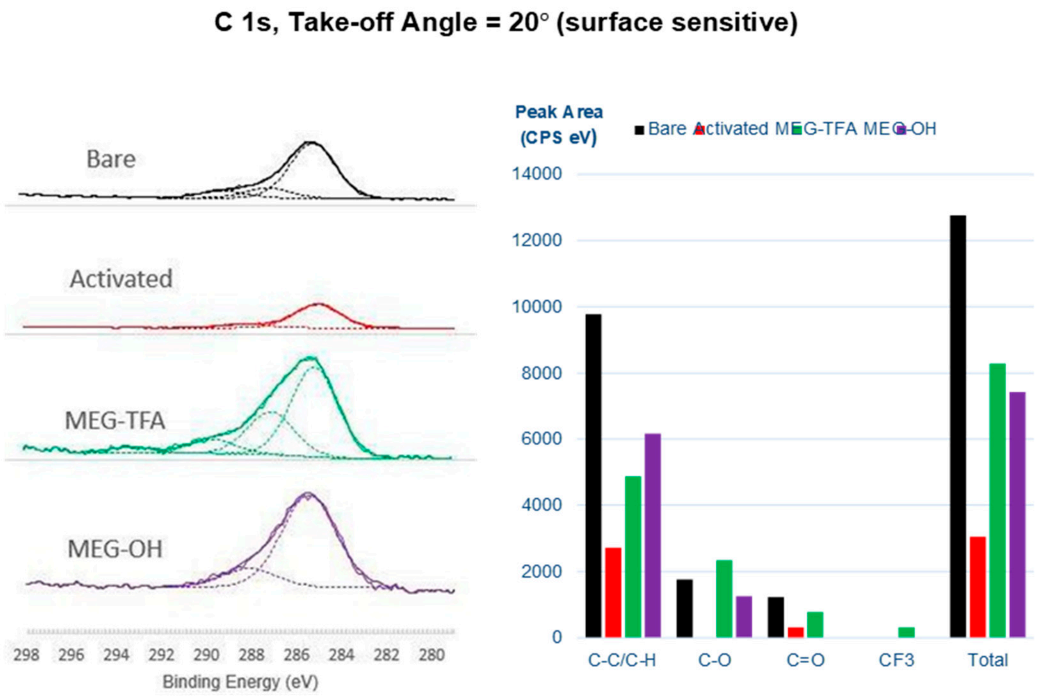

Figure S2. C 1s, Take-off Angle = 20° (surface sensitive).

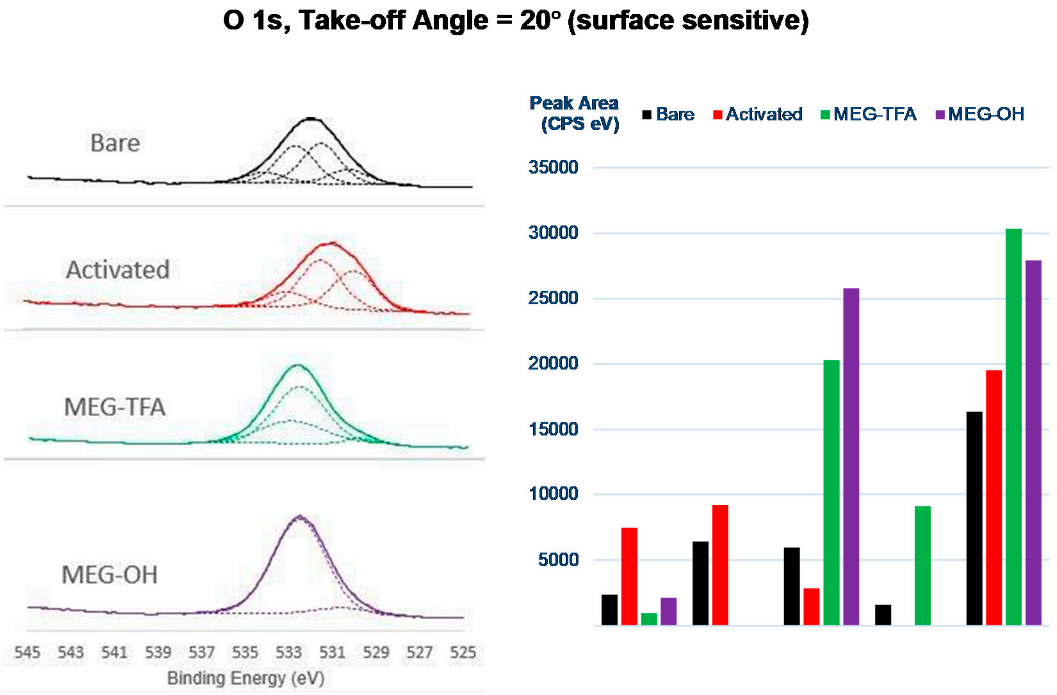

Figure S3. O 1s, Take-off Angle = 20° (surface sensitive).

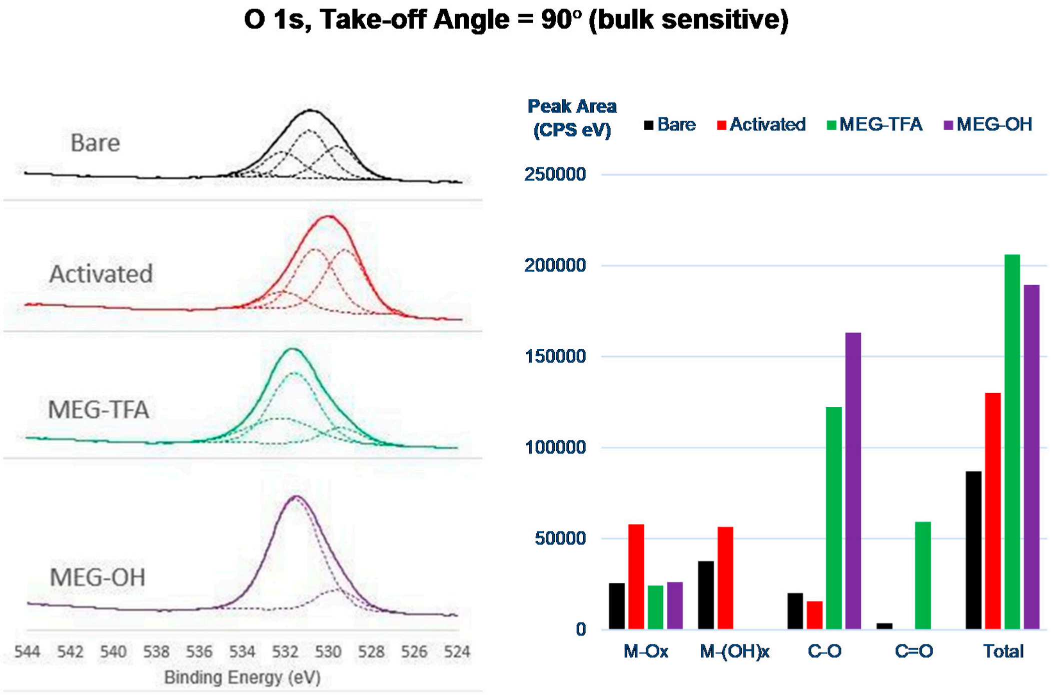

Figure S4. O 1s, Take-off Angle = 90° (bulk sensitive).

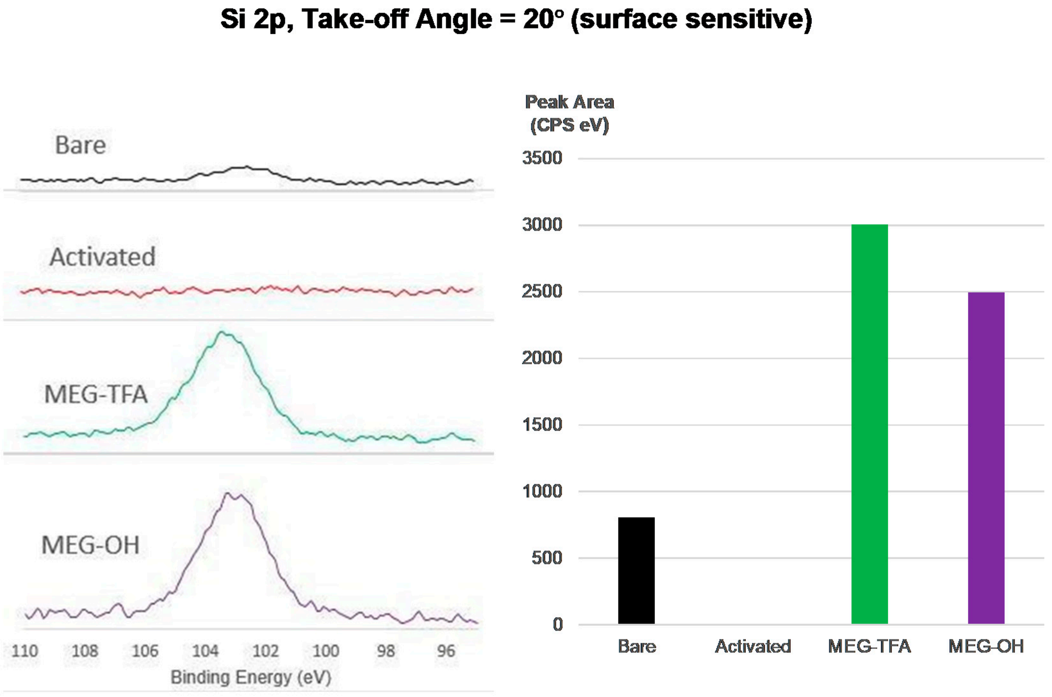

Figure S5. Si 2p, Take-off Angle = 20° (surface sensitive).

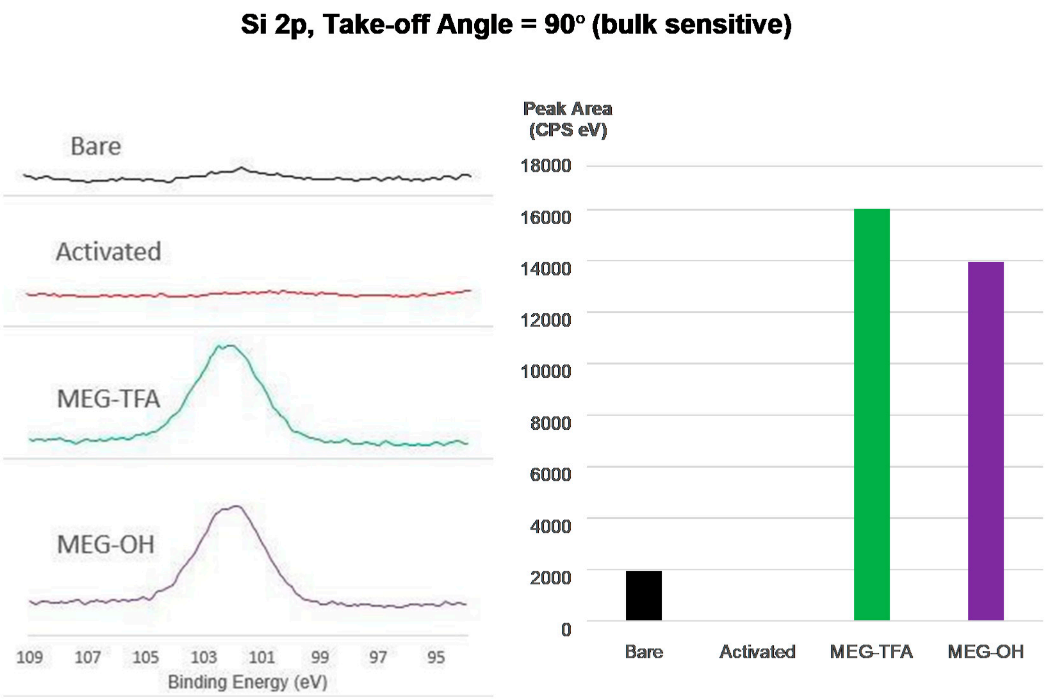

Figure S6. Si 2p, Take-off Angle = 90° (bulk sensitive).

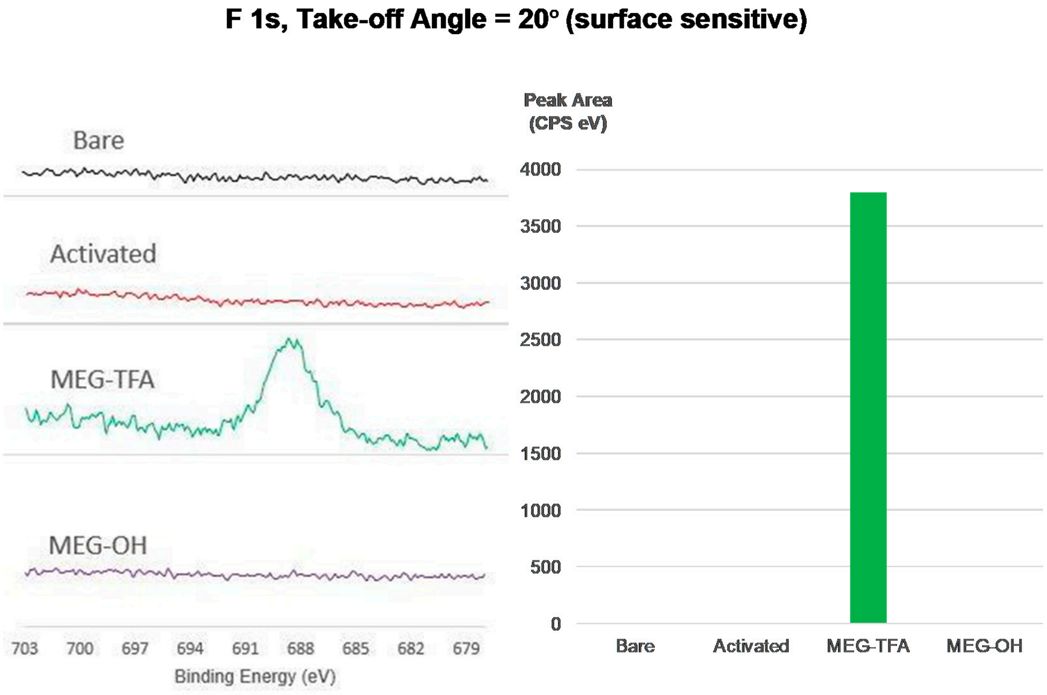

Figure S7. F 1s, Take-off Angle = 20° (surface sensitive).

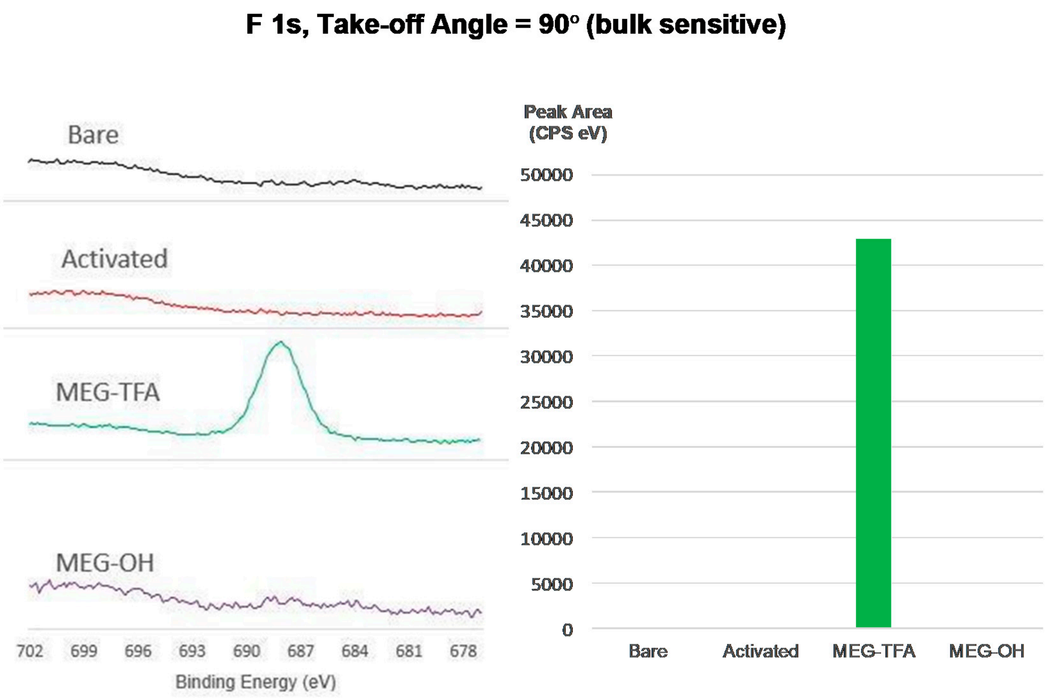

Figure S8. F 1s, Take-off Angle = 90° (bulk sensitive).

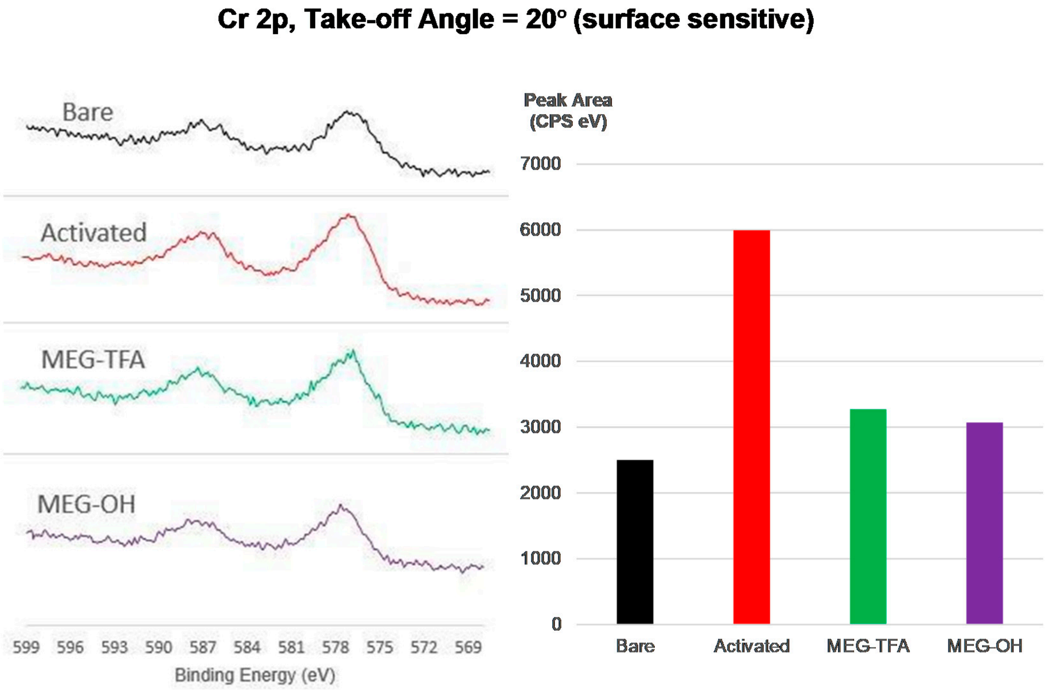

Figure S9. Cr 2p, Take-off Angle = 20° (surface sensitive).

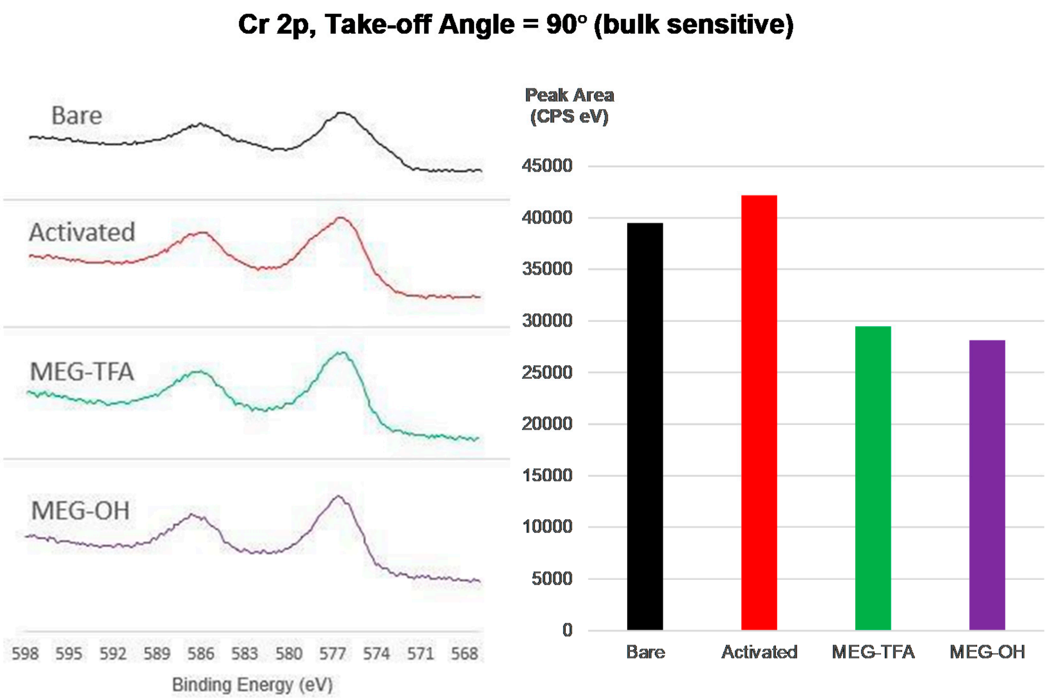

Figure S10. Cr 2p, Take-off Angle = 90° (bulk sensitive).
